# *D*-BMAP18 Antimicrobial Peptide Is Active *In vitro*, Resists to Pulmonary Proteases but Loses Its Activity in a Murine Model of *Pseudomonas aeruginosa* Lung Infection

**DOI:** 10.3389/fchem.2017.00040

**Published:** 2017-06-19

**Authors:** Mario Mardirossian, Arianna Pompilio, Margherita Degasperi, Giulia Runti, Sabrina Pacor, Giovanni Di Bonaventura, Marco Scocchi

**Affiliations:** ^1^Department of Life Sciences, University of TriesteTrieste, Italy; ^2^Department of Medical, Oral, and Biotechnological Sciences, Università degli Studi “G. d'Annunzio“ Chieti-PescaraChieti, Italy; ^3^Center of Excellence on Aging and Translational Medicine (CeSI-MeT), “G. d'Annunzio” University FoundationChieti, Italy

**Keywords:** antimicrobial peptide, BMAP18, cystic fibrosis, *Pseudomonas aeruginosa*, proteolytic degradation, bronchoalveolar lavage, lung

## Abstract

The spread of antibiotic resistant-pathogens is driving the search for new antimicrobial compounds. Pulmonary infections experienced by cystic fibrosis (CF) patients are a dramatic example of this health-care emergency. Antimicrobial peptides could answer the need for new antibiotics but translating them from basic research to the clinic is a challenge. We have previously evaluated the potential of the small membranolytic peptide BMAP-18 to treat CF-related infections, discovering that while this molecule had a good activity *in vitro* it was not active *in vivo* because of its rapid degradation by pulmonary proteases. In this study, we synthesized and tested the proteases-resistant all-*D* enantiomer. In spite of a good antimicrobial activity against *Pseudomonas aeruginosa* and *Stenotrophomonas maltophilia* clinical isolates and of a tolerable cytotoxicity *in vitro, D*-BMAP18 was ineffective to treat *P. aeruginosa* pulmonary infection in mice, in comparison to tobramycin. We observed that different factors other than peptide degradation hampered its efficacy for pulmonary application. These results indicate that *D*-BMAP18 needs further optimization before being suitable for clinical application and this approach may represent a guide for optimization of other anti-infective peptides eligible for the treatment of pulmonary infections.

## Introduction

Cystic fibrosis (CF) is a genetic disorder that significantly reduces the life expectancy. Most of CF-patients succumb to respiratory failure brought on by chronic bacterial infection and airway inflammation (Cribbs and Beck, 2017). CF lung disease begins early in life with inflammation and impaired muco-ciliary clearance, and gets worse due to the consequent chronic infection of the airways (Robinson and Bye, 2002). A progressive decline of lung function then follows, with episodes of acute aggravation of respiratory symptoms. CF has a peculiar set of bacterial pathogens that are frequently acquired in an age-dependent sequence (Gibson et al., 2003) and *Pseudomonas aeruginosa* represents the most problematic-one, infecting 60–75% of the patients (Ahlgren et al., 2015). Moreover, physicians treating patients with CF are increasingly faced with infections by multidrug-resistant isolates of *P. aeruginosa* and other pathogens (Dasenbrook et al., 2010; Wu et al., 2016). The problem is also exacerbated by the high microbial adaptation to the CF pulmonary environment, resulting in an increased ability to form biofilms intrinsically resistant to antibiotics (Bhagirath et al., 2016). Multidrug-resistant infections may be treated successfully by using combinations of antibiotics with different mechanisms of action. Unfortunately, very few novel antimicrobials have come out in the last years to complement current therapies (Harbarth et al., 2015). Furthermore, potential new antibiotic drugs should be supported by other, non-classical, antimicrobial agents, in order to contain the diffusion of pathogens resistant even to these new compounds (Waters and Smyth, 2015). Anti-Microbial Peptides (AMPs) are under the spotlight as a promising class of antimicrobials for development as novel antibiotics (Mahlapuu et al., 2016). They are naturally occurring molecules of the innate immune system of animals with important roles in host defense (Lai and Gallo, 2009; Yeung et al., 2011). Most AMPs have a wide spectrum of activity also comprising multidrug-resistant pathogens; a relatively good selectivity toward bacteria and a rapid mechanism of action, most often based on the lysis/permeabilization of microbial membranes. This mode of action, in which no specific molecular targets are involved, is associated with a low frequency for selection of resistant strains (Brogden, 2005; Benincasa et al., 2006; Hancock and Sahl, 2006). The antimicrobial activity of AMPs has also been largely reported, with respect to CF pathogens (Saiman et al., 2001; Zhang et al., 2005; Kapoor et al., 2011; Bezzerri et al., 2014; Mangoni et al., 2015). Previously, we showed that some bovine alpha-helical AMPs [the BMAPs Skerlavaj et al., 1996] had a potent and rapid *in vitro* bactericidal and anti-biofilm activity against many *P. aeruginosa* and *S. maltophilia* strains from CF patients (Pompilio et al., 2011, 2012). We also demonstrated that some N-terminal shortened fragments of these peptides overall maintained their good antibacterial properties toward CF-related pathogens, but show a certain degree of acute toxicity when intra-tracheally administered to mice lungs. BMAP27(1–18) was selected for its good antimicrobial potential and reduced pulmonary toxicity, but its protective effect against *P. aeruginosa* lung infection in mice was scarce due to its rapid degradation in the pulmonary environment (Mardirossian et al., 2016). Proteolytic digestion is a problem that BMAP27(1–18) shared with many other native (Moncla et al., 2011; Mattiuzzo et al., 2014) and synthetic (Kim et al., 2014) antimicrobial peptides, reported also in the pulmonary environment (Sajjan et al., 2001; Morris et al., 2012). The use of enantiomeric all *D*-peptides represented a promising strategy to avoid proteolysis in lungs (Sajjan et al., 2001) and could possibly enhance BMAP27(1–18) antibacterial activity *in vivo*, reducing its degradation in the pulmonary environment. In this work, we synthesized the *D*-isomer of BMAP27(1–18), *D*-BMAP18. We tested *D*-BMAP18 it for stability in bronchoalveolar lavage (BAL) fluid, *in vitro* antibacterial activity, *in vivo* protective effect, and toxicity both *in vitro* and *in vivo*.

## Materials and methods

### Bacterial strains

*P. aeruginosa, S. maltophilia*, and *Staphylococcus aureus* strains were originally isolated from respiratory specimens of CF patients admitted to the “Bambino Gesù” Children Hospital in Rome and previously tested with AMPs (Pompilio et al., 2012). *P. aeruginosa* RP73, and PAO1 were tested as reference strains.

### Design and synthesis of *D*-BMAP18

The peptide *D*-BMAP18 (GRFKRFRKKFKKLFKKLS-am) was synthesized using the solid-phase Fmoc chemistry on a microwave peptide synthesizer Astra Initiator + (Biotage, USA). Protected amino acids and Fmoc-linker-AM champion resins were purchased from Advanced Biotech Italia (Milan, Italy) or Novabiochem (Merck, Darmstadt, Germany). For each coupling step, the Fmoc-protected amino acid and coupling reagents were added in a 5-fold molar excess with respect to resin substitution. Couplings were carried out with N-hydroxybenzotriazole (HOBt) and 2-(1H-benzotriazole-1-yl)-1,1,3,3-tetramethyluronium tetrafluoro-borate (TBTU) at 75°C. Cleavage from the resin and deprotection of the synthesized peptide were carried out with a solution of 85% trifluoroacetic acid (TFA), 2% water, 2% triisopropylsilane and 8% of phenol, 1,2-ethanedithiol and 3% of thioanisole. The peptide was purified by reversed phase HPLC on a C18 column (19 × 300 mm; Waters, MA, USA) using 0–40% acetronitrile-water linear gradients in 0.05% trifluoroacetic acid. Peptide's quality and purity was verified by ESI–MS (API 150 EX Applied Biosystems), *(D*-BMAP18 theoretical average mass = 2341.95 Da; measured average mass = 2342.2 Da). The peptide was lyophilized from 10 mM HCl solution to remove TFA and the concentration of the stock solution was evaluated by spectrophotometric determination of peptide bonds (ε_214_) (Kuipers and Gruppen, 2007).

### *D*-BMAP18 degradation in bronchoalveolar lavage (BAL) fluid

Bronchoalveolar fluid was collected from six C57/Bl6NCrl healthy male mice (2–3 months old, from the animal facility of the University of Trieste). Mice were killed by cervical dislocation, a blunt needle connected to a syringe was inserted into the mouth and trachea, and then lungs were washed with 1 ml of sterile, pre-warmed (37°C), 0.9% NaCl. Equal volumes of BAL samples from each mouse were pooled and stored in aliquots at −20 °C until further uses. The total protein concentration of BAL fluid (300 μg/ml) was determined by the BCA assay (Pierce, BCA Protein Assay Kit). To evaluate *D*-BMAP18 resistance to proteases, a very small volume of a highly concentrated solution of peptide was diluted in pooled BAL to a final concentration of 300 μg/ml, reaching a 1:1 (wt/wt) peptide/BAL total proteins ratio. *D*-BMAP18 in BAL was then incubated at 37°C. Thirty microliters of the mixture were sampled at indicated times, immediately cooled down on ice and frozen at −20°C. Subsequently, samples were heated for 5 min at 90°C in Laemmli Sample Buffer A, and 10 μl of each sample were loaded on a 16% tricine gel for SDS-PAGE (Schagger, 2006). The gel was stained over-night with Coomassie Brilliant Blue and destained with 10% acetic acid in water (v/v). These experiments were carried out in accordance with the recommendations of the Guide for the Care and Use of Laboratory Animals of the National Institute of Health and in accordance with the ethical standards of the Animal Care Committee of the University of Trieste. The protocol was approved by the Ethics Committee of the University of Trieste.

### Evaluation of antibacterial activity of *D*-BMAP18

MIC values were determined using the broth microdilution method. Briefly, serial 2-fold dilutions of each peptide were prepared in Mueller-Hinton broth (MH; Difco) and aliquoted in round-bottom 96 well microtiter plates (Sarstedt). Each well was inoculated with a standardized inoculum to achieve a final test concentration of about 5 × 10^5^ CFU/ml. The MIC was measured as the lowest concentration of the peptide that completely inhibited visible bacterial growth after incubation at 37°C for 24 h. To calculate the MBC, following the 24 h-incubation of the MIC assay, 25 μl of broth from clear wells were plated on MH agar plates, and incubated at 37°C for 24 h. MBC was defined as the lowest concentration of the peptide killing at least 99.9% of the original inoculum.

### *In vitro* toxicity of *D*-BMAP18 against cell lines

Cytotoxicity was determined by the MTT assay using human lung carcinoma A-549 cells (German DMSZ). Cells were grown, at 37°C and 5% CO_2_, to sub-confluence in 100 μL of Dulbecco's MEM (Sigma-Aldrich) + 5% FBS (Euroclone) + 2.4 mM Glucose + 1% Pen/Strep, using a 96 wells flat-bottom microtiter plates. Serial 2-fold dilutions of the peptide were prepared in cell growth medium and 100 μL were added to the cells. After a 24 h-incubation the cells were washed using PBS (Sigma-Aldrich), 100 μL of PBS were added and 10 μL MTT were then added (5 mg/ml in PBS, Sigma-Aldrich). Following 4 h-incubation, 150 μl of IGEPAL 10% in 0.01N HCl were added (Sigma-Aldrich) and the plates were incubated at 37°C. After over-night incubation, the cytotoxicity was spectrophotometrically evaluated by measuring OD_620nm_.

### *In vivo* toxicity of *D*-BMAP18

*In vivo* acute toxicity of *D*-BMAP18 was evaluated in C57BL/6NCrl mice (*n* = 5/group) (male; 22 g; 6 ± 2 week-old) obtained from Charles River Laboratories Italia S.r.l. (Calco, Milan, Italy). Mice were intra-tracheally challenged with increasing doses (1, 2, 4, and 8 mg/kg) of *D*-BMAP18 prepared in sterile distilled water. Control mice received vehicle only (sterile distilled water). General health and animal behavior (ruffled coats, huddled position, lack of retreat in handler's presence), weight loss, and survival were monitored daily over a 5-day period, comparatively to control mice. Mice were sacrificed 5 days post-exposure (p.e.) by intraperitoneal injection of tribromoethanol (Sigma-Aldrich), then lungs were observed *in situ* for macroscopic damage using the “four-point scoring system” (Johansen et al., 1993): 1, normal; 2, swollen lungs, hyperemia, and small atelectasis; 3, pleural adhesion, atelectasis, and multiple small abscesses; and 4, large abscesses, large atelectasis, and hemorrhages. Subsequently, lungs were removed *en bloc* from the chest via sterile excision and immediately weighed. These experiments were carried out in accordance with the recommendations of the Guide for the Care and Use of Laboratory Animals of the National Institute of Health and in accordance with the ethical standards of the Animal Care Committee of the “G. d'Annunzio” University of Chieti-Pescara. The protocol was approved by the Animal Care Committee of the “G. d'Annunzio” University of Chieti-Pescara.

### Activity of *D*-BMAP18 against mouse acute lung infection caused by *P. aeruginosa*

C57/Bl6NCrl mice (*n* = 8/group) were intratracheally challenged with 1 × 10^7^ cells *P. aeruginosa* RP73 clinical strain, and 5 min later a single dose of *D*-BMAP18 at different concentrations (0.5, 1, and 2 mg/kg) was intratracheally administrated. Sterile distilled water alone or tobramycin [10 mg/kg] (Sigma-Aldrich S.r.l.), were used respectively as negative and positive control. One day post-exposure, mice were sacrificed by intraperitoneal injection of tribromoethanol (Sigma-Aldrich S.r.l.), then lungs were observed *in situ* for macroscopic damage (Johansen et al., 1993), aseptically removed *en bloc* from the chest, and immediately weighed. Subsequently, lungs were homogenized (24,000 rpm) on ice in 1 ml of sterile PBS by using Ultra-Turrax T25-Basic homogenizer (IKA-Werke GmbH & Co. KG, Germany). Ten-fold serial dilutions of lung homogenates were plated on MH agar (Oxoid SpA), and the number of colony-forming units (CFU) was counted after incubation at 37°C for 24 h.

These experiments were carried out in accordance with the recommendations of the Guide for the Care and Use of Laboratory Animals of the National Institute of Health and in accordance with the ethical standards of the Animal Care Committee of the “G. d'Annunzio” University of Chieti-Pescara. The protocol was approved by the Animal Care Committee of the “G. d'Annunzio” University of Chieti-Pescara.

### Evaluation of antibacterial activity of *D*-BMAP18 in BAL

The antimicrobial activity of *D*-BMAP18 in the presence of BAL from healthy mice was evaluated against *P. aeruginosa* RP73 strain by a killing assay. Sterile 0.9% w/v NaCl alone was used as a control. Different concentrations of the peptide were prepared in 100 μL of BAL. To each concentration was then added 100 μL of bacterial suspension prepared in MH broth and diluted in sterile 0.9% w/v NaCl to a load of 2 × 10^6^ CFU/ml. The final conditions were: 10^6^ CFU/ml *P. aeruginosa* RP73 in 50% BAL in sterile 0.9% w/v NaCl (v/v). The final % of residual MH medium introduced in the assay with the diluted bacteria was below 5%. Samples were then incubated at 37°C for 1 h, serially 10-folds diluted in MH broth and plated on MH agar. Colonies were counted after overnight incubation at 37°C.

### Statistical analysis

Statistical analysis of results was conducted with GraphPad Prism version 4.00 (GraphPad software Inc.; San Diego, CA, USA), considering as statistically significant a *p*-value < 0.05. Parametric (ANOVA-test followed by Student-Newmann-Keuls post-test) or non-parametric (Kruskal-Wallis test followed by Dunn's multiple comparison test) tests were performed when data were normally distributed or not, respectively. Differences in MIC values were considered statistically significant if > 2 log_2_.

## Results

### *D*-BMAP18 stability in murine BAL fluid

**Figure 1:**
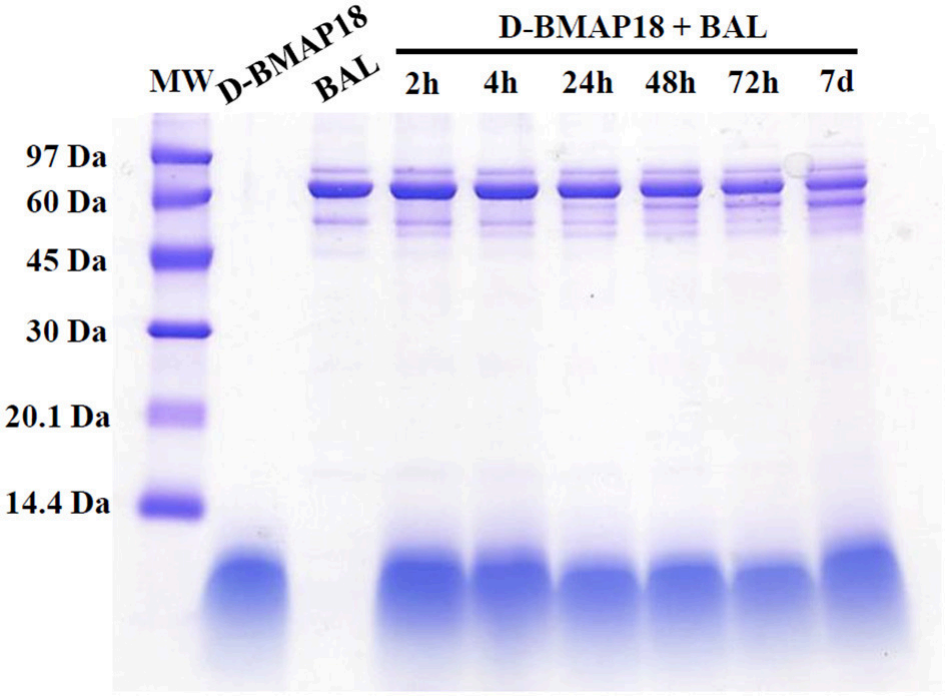
Evaluation of the stability of *D*-BMAP18 in the presence of murine bronchoalveolar lavage (BAL) fluid at 37°C. Samples were collected at prefixed times and analyzed by SDS-PAGE (gel 16%, tricine) following staining with Coomassie Brilliant Blue. As control, 2.4 μg of *D*-BMAP18 and BAL alone were loaded, corresponding to the original concentration of both compounds at the beginning of the time-course.

We recently showed that *L*-BMAP-18 peptide is degraded by pulmonary proteases in murine bronchoalveolar lavage fluids within 20 min of exposure, and already after 10 min most of the L-BMAP-18 was digested (see Mardirossian et al., 2016). *D*-BMAP18 was synthesized to provide a molecule more resistant to enzymatic cleavage. The stability of *D*-BMAP18 was tested in murine BAL fluid. No peptide degradation was in fact observed, even after 7 days of incubation at 37° C in undiluted BAL (Figure 1), indicating that *D*-BMAP18 is not a substrate for proteases in murine BAL.

### *D*-BMAP18 *in vitro* antibacterial activity

We evaluated the *in vitro* antimicrobial activity of *D*-BMAP18 against multiresistant CF isolates of *P. aeruginosa, S. maltophilia* and *S. aureus*, previously tested with *L*-BMAP18 (Mardirossian et al., 2016). Overall, *D*-BMAP18 showed a relevant activity against both *P. aeruginosa* and *S. maltophilia* strains, whereas no effect was observed toward *S. aureus* (Table 1). Compared to the *L*-isomer, *D*-BMAP18 exhibited comparable activity against *P. aeruginosa* (MIC_90_: 16 μg/mL, for both AMPs), but significantly higher activity toward *S. maltophilia* (MIC_90_: >32 and 16 μg/ml, respectively). In particular, MIC values indicated that *D*-BMAP18 was significantly more active than L-isomer against *P. aeruginosa* RP73 and PA10, and *S. maltophilia* SM122. MBC/MIC ratio (killing quotient, KQ) was ≤ 4 for most of strains, indicating the bactericidal activity of both peptides.

**Table 1:** Antibacterial activity of *L*-BMAP18 and *D*-BMAP18 against Gram-positive and Gram-negative strains from CF patients.

	***L*-BMAP17 (μg/ml)**			***D*-BMAP18 (μg/ml)**		
**Strains**	MIC^a^^*^	MBC^a^^*^	**KQ^b^**	**MIC^a^**	**MBC^a^**	**KQ^b^**
***P. aeruginosa***						
PA01	8	8	1	8	32	4
RP73	32	32	1	4	8	2
PA03	8	16	2	8	8	1
PA05	4	8	2	4	8	2
PA07	4	8	2	8	8	1
PA08	8	>32	>4	8	16	2
PA09	16	>32	>2	16	32	2
PA10	>32	>32	−	16	32	2
PA14	8	32	4	16	32	2
PA21	8	32	4	16	32	2
PA22	2	8	2	4	32	8
PA31	8	16	2	16	32	2
***S. maltophilia***						
SM103	4	8	2	4	4	1
SM105	8	>32	>4	8	8	1
SM106	>32	>32	−	32	32	1
SM110	4	8	2	4	4	1
SM120	16	>32	>2	8	16	2
SM122	>32	>32	−	16	16	1
SM123	32	32	1	16	16	1
SM126	32	>32	>1	16	32	2
SM130	8	8	1	8	16	2
SM136	8	8	1	8	16	2
SM139	8	16	2	8	8	1
SM143	4	16	4	8	8	1
***S. aureus***						
SA1	>32	−	−	>32	−	−
SA2	>32	−	−	>32	−	−
SA3	>32	−	−	>32	−	−
SA4	32	−	−	>32	−	−
SA5	32	−	−	>32	−	−
SA7	32	−	−	>32	−	−

*Minimum inhibiting (MIC) and minimum bactericidal (MBC) concentrations are shown. Results are from three independent experiments performed as internal duplicates (n = 6)*.

* *These data were already published (Mardirossian et al., 2016)*.

a *MIC and MBC values are expressed as μg/ml*.

b *KQ, killing quotient, measured as MBC/MIC ratio: KQ ≤ 4 is suggestive for bactericidal effect, KQ > 4 is suggestive for bacteriostatic effect*.

### *In vitro D*-BMAP18 cytotoxicity

**Figure 2:**
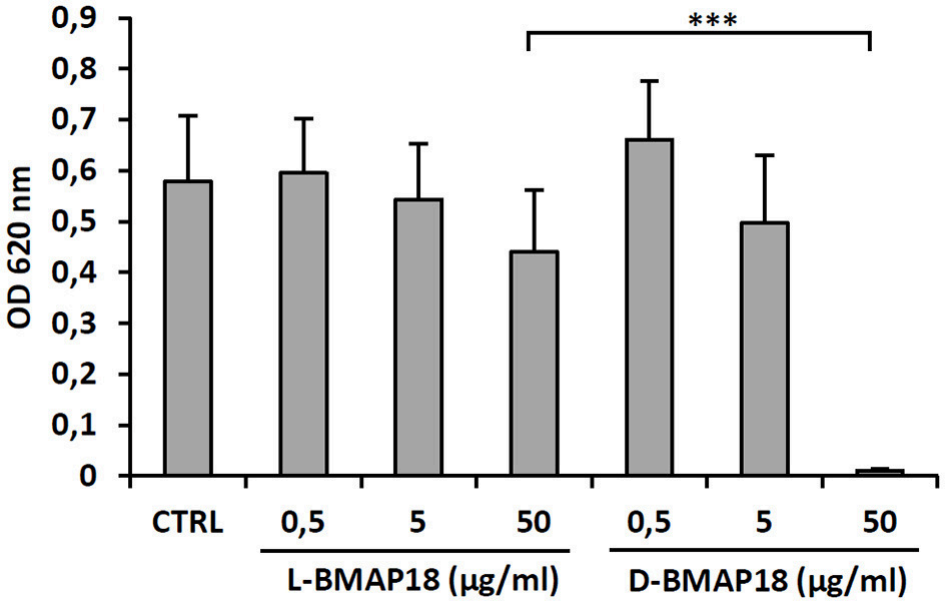
Cytotoxicity of *L*- and *D*-BMAP18 toward A-549 human pulmonary cells, as assessed by MTT assay, cells following 24 h-exposure to *L*- and *D*-BMAP18. In control samples (CTRL), cells were exposed to the vehicle only. Results, from at least two independent experiments performed as internal triplicate (*n* ≥ 6), are shown as mean + *SD*. ^***^p < 0.001, ANOVA + Student-Newmann-Keuls post-test.

Cytotoxicity of *D*-BMAP18 and *L*-BMAP18 was evaluated, by the MTT assay against human pulmonary A-549 epithelial cells, to simulate the toxicity toward the host pulmonary cells. Both peptides did not significantly affect cell viability at a concentration of 5 μg/ml (Figure 2) and became cytotoxic only at 50 μg/ml. At this concentration, *D*-BMAP18 was, unexpectedly, more cytotoxic than the *L*-isomer (Figure 2), probably because the *L*-form of the peptide was more easily degraded by extracellular proteases secreted by cells, and therefore its cytotoxic effect was lower.

### *In vivo* acute toxicity of *D*-BMAP18

**Figure 3:**
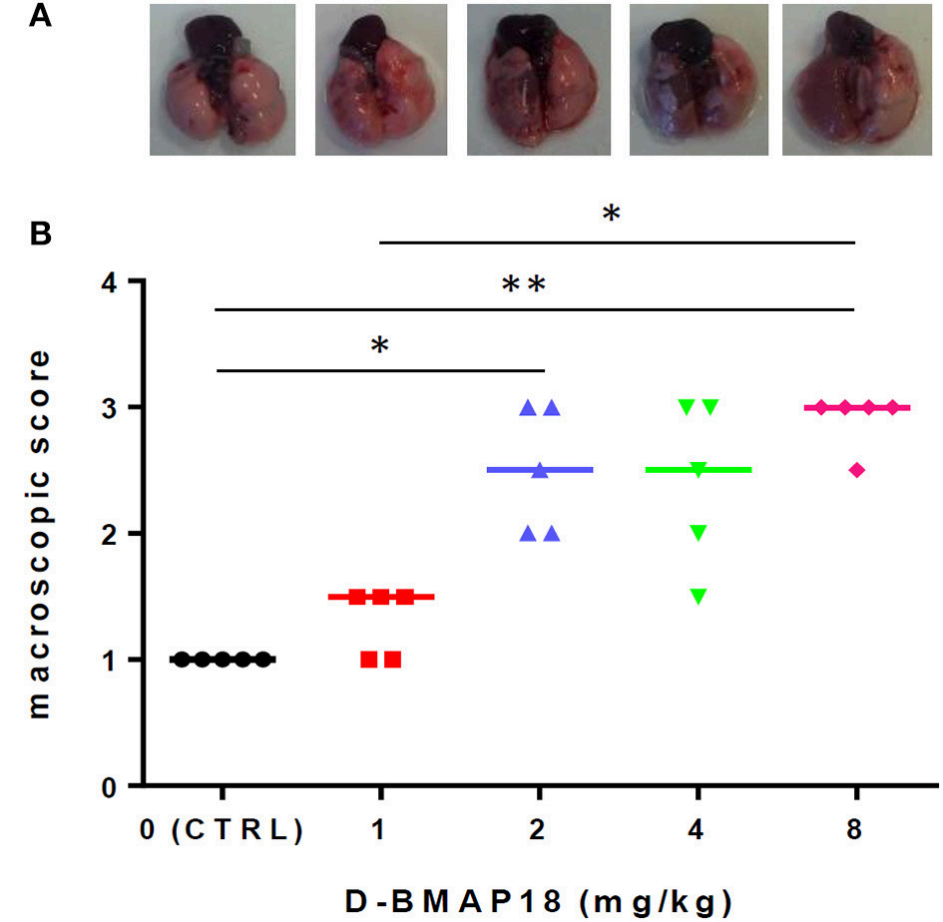
*In vivo* toxicity study: macroscopic damage in C57BL/6NCrl mouse lungs (*n* = 5/group) following a single exposure to *D*-BMAP18 (1, 2, 4, and 8 mg/kg), or to vehicle (H_2_O) only (CTRL). **(A)** Representative lung photograph for each condition are shown. **(B)** Macroscopic lung pathology was assessed on day 5 p.e. by using a “four-point scoring system” (Johansen et al., 1993): (1) normal; (2) swollen lungs, hyperemia, and small atelectasis; (3) pleural adhesion, atelectasis, and multiple small abscesses; and (4) large abscesses, large atelectasis, and hemorrhages. Horizontal bars are median values. Representative photographs for each condition are shown. ^*^p < 0.05, ^**^p < 0.01, Kruskal-Wallis + Dunn's multiple comparison post-test.

We assessed the toxicity of *D*-BMAP18 in C57BL/6NCrl mice after a single intratracheal instillation of peptide at increasing concentrations. Macroscopic lung pathology was assessed on day 5 post exposure by using a “four-point scoring” system (Figure 3). No pulmonary damage was observed in unexposed or 1 mg/kg-treated mice, as assessed by the macroscopic score index evaluation. On the contrary, exposure to 2 and 8 mg/kg doses significantly damaged lungs (median score: 2.5 vs. 3; *p* < 0.05 and *p* < 0.01, respectively), as indicated by extensive hyperemia, atelectasis, and pleural adhesion.

Changes in mice and pulmonary weight (Figures S1A,B) were measured over 5 days p.e. Exposure to the peptide caused significant reduction in body weight also at the lowest dose, and this effect is dose-dependent. Variations in pulmonary weight confirmed the same trend found for macroscopic score analysis.

### *In vivo* protective effect of *D*-BMAP18

**Figure 4:**
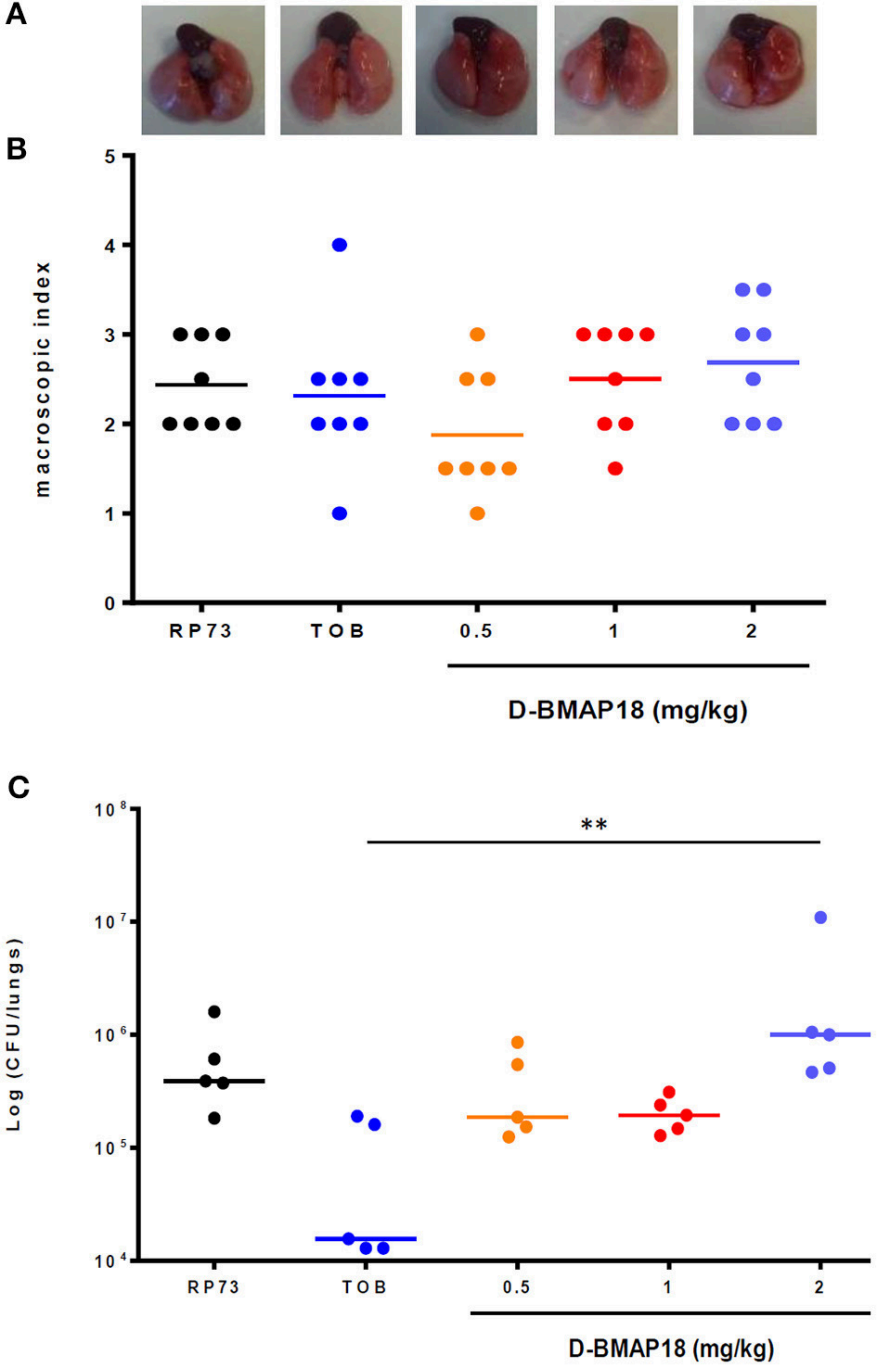
*In vivo* protection study: macroscopic damage and bacterial load of C57BL/6NCrl mouse lungs measured on day 1 following *P. aeruginosa* RP73 infection. After infection, mice (*n* = 8/group) were administered with *D*-BMAP18 (0.5, 1, and 2 mg/kg) or Tobramycin (TOB) 10 mg/kg. Mice challenged with *P. aeruginosa* RP73, but not treated, were used as control group. **(A)** Representative lungs photographs for each condition are shown. **(B)** Macroscopic lung pathology was assessed on day 1 p.e. by using a “four-point scoring system” (Johansen et al., 1993) (see Figure 3). For each group of mice, the lung of 3 mice out of 8 were fixed to perform future histological analyses. Horizontal bars are median values (*n* = 8/group). Kruskal-Wallis + Dunn's multiple comparison test showed no significant differences. **(C)** Lungs of the remaining mice (5 out of 8) were homogenized and plated onto MH agar to assess viable bacteria. Bacterial colony counts from each mouse were expressed as CFU/lungs. Horizontal bars are median values. ^**^p < 0.01, Kruskal-Wallis + Dunn's multiple comparison post-test.

We assessed the *in vivo* antimicrobial potential of *D*-BMAP18 in a murine model of acute pulmonary infection caused by *P. aeruginosa*. On day 1 post-infection, mice and mice lungs weight, as well as macroscopic lung pathology and pulmonary bacterial load were assessed (Figure S2 and Figure 4).

No significant differences in mice body weight were observed on day 1 post-exposure, regardless of the group considered (not shown). A positive trend was found between *D*-BMAP18 dose and macroscopic damage, although it was not statistically significant because of the relevant data dispersion (Figure 4B). The lungs of mice treated with *D*-BMAP18 at 1 and 2 mg/kg were slightly more edematous, as suggested by the observed weight, compared to those administrated with tobramycin 10 mg/kg (Figure S2). The administration of *D*-BMAP18 was not protective against *P. aeruginosa* RP73 infection, regardless of the considered doses. The bacterial load measured in mice treated with 2 mg/kg *D*-BMAP18 was significantly higher than that observed in tobramycin-treated lungs (Figure 4C). Mortality was observed only in one tobramycin-treated mouse (1 out of 8; 12.5%).

### Antibacterial activity of *D*-BMAP18 in presence of BAL fluid

**Figure 5:**
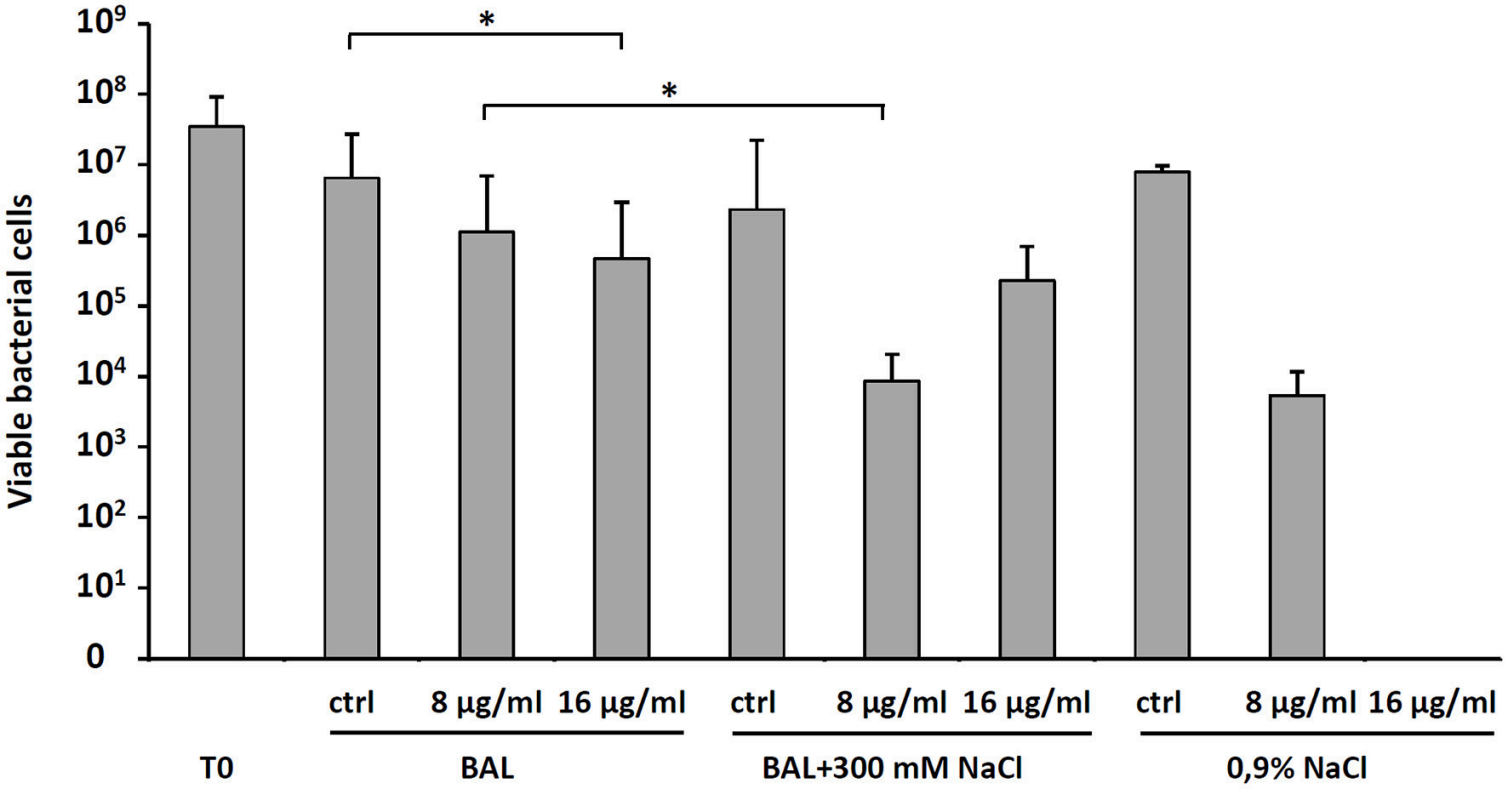
Effect of bronchoalveolar lavage (BAL) without or with the addition of 300 mM NaCl, or 0.9% NaCl on the *D*-BMAP18 activity against *P. aeruginosa* RP73 strain. Peptide has been used at 8 and 16 ug/ml and bacterial killing was evaluated by viable cell count. In control samples, *P. aeruginosa* RP73 was exposed at H_2_O only. Results are expressed as mean values + SDs (*n* = 6). ^*^p < 0.05, ANOVA + Student-Newmann-Keuls post-test.

To explain the moderate *in vivo* antibacterial activity of *D*-BMAP18 despite its increased stability to proteolysis, to exclude procedural mistakes during *in vivo* assays, and to exhaustively evaluate its antimicrobial efficacy as a drug intended for pulmonary applications, we looked for conditions roughly approximating the lung environment used for *in vivo* experiments. Therefore, we performed a bacterial killing assay on *P. aeruginosa* RP73 incubating bacteria and peptide in presence of BAL fluid at 37°C. As a control, we used sterile 0.9% (w/v) NaCl, since it was used to wash the murine lung for collecting BAL. We observed that BAL fluid decreased the antimicrobial efficacy of 16 μg/ml *D*-BMAP18, and abolished its activity when used at 8 μg/ml (Figure 5). We hypothesized that this inhibition may be due to peptide binding/sequestration by BAL components, given its stability under the tested conditions. When the assay was repeated after adding 300 mM NaCl to the medium, the antimicrobial activity was partially restored (Figure 5) suggesting that electrostatic interaction between the components of BAL and the peptide may play a role in the inhibition.

## Discussion

The bacterial pathogens causing CF lung infections represent a life threat for patient because of their resistance to antibiotics, and because of their adaptation to the peculiar characteristic of CF-lungs. Antimicrobial peptides may potentially be effective compounds to combat pulmonary infections, but some aspects of their use need to be further analyzed and eventually optimized for future applications (Marr et al., 2006). In a previous study, we demonstrated that BMAP18 has a promising antibacterial activity against both *P. aeruginosa* and *S. maltophilia*. However, the peptide is rapidly degraded in murine pulmonary fluid, suggesting it may be unstable also in human lungs (Mardirossian et al., 2016). In this study, to overcome the degradation problems, we synthesized and tested an all-D isomer of BMAP18. The artificial introduction of *D*-amino acids in peptides has been widely used to avoid proteolysis in biological samples (Sajjan et al., 2001; Hamamoto et al., 2002). Moreover, the presence of single *D*-aminoacids into the structure of L-antimicrobial peptides has been shown to affect their structuring as α-helices. These modifications reduced on the one hand their activity on the cellular membrane, and therefore their cytotoxicity, but on the other hand, also their efficacy to bind to and insert into the LPS layer of the bacterial outer membrane, therefore decreasing their antimicrobial potency (Ghosh et al., 2016). We showed that *D*-BMAP18 is a possible alternative to the *L*-form to avoid proteolysis without losing its antibacterial potential. Our results in fact indicate that *D*-BMAP18 is stable when exposed to protease-rich BAL fluid from mice and, most importantly, its antimicrobial activity is comparable with that exhibited by the *L*-isomer. It is worth noting that often the MBC of both *D*-BMAP18 and *L*-BMAP18 is only twice the MIC, indicating that these AMPs possess a bactericidal activity. This makes these peptides even more attractive for the treatment of persistent infections. Interestingly, the *D*-isomer exhibited higher activity against *P. aeruginosa* RP73 and PA10 strains compared to the L-isomer. It is believed that *D*-analogs of membranolytic alpha helical AMPs are equipotent to the naturally occurring all-L peptides (Wade et al., 1990; Giangaspero et al., 2001). We therefore think that the different MICs we observed for RP73 and PA10 strains could be due to the different capacity of strains to inactivate or degrade the L-form. We also showed that the *D*-peptide, *in vitro*, is not toxic up to 5 μg/ml against human pulmonary A-549 epithelial cells. However, at higher concentrations (50 μg/ml), the *D*-BMAP18 becomes more toxic than the L-form. This additional toxicity may be linked to the failure of eukaryotic cells to selectively degrade *D*-peptide during the incubation, but we cannot exclude other reasons. A certain toxic effect of *D*-BMAP18 was confirmed also by acute toxicity test in lungs of mice, showing a concentration-dependent trend. We think that the route of administration could contribute to the toxicity. In fact the intra-tracheal administration route temporarily exposes narrow regions of the lung tissues to high, and presumably toxic, peptide concentrations. This effect could mask the beneficial antibacterial activity of the molecule. Microaerosol administration, or alternative devices, could be considered for further assays (Valenti et al., 2017). As a further strategy to decrease the *D*-BMAP18 cytotoxicity, is now under investigation its administration as a cleavable pro-drug, in order to guarantee a controlled release of the drug. This stratagem could maintain the peptide's potent antimicrobial effect while mitigating the *D*-BMAP18 toxic effects on the pulmonary epithelium.

In light of the *in vitro* activity against *P. aeruginosa* and the acute *in vivo* toxicity, we assumed to have a sufficient therapeutic window to test the effectiveness of the peptide against a murine *P. aeruginosa* acute pulmonary infection. We considered concentrations around 1 mg/kg as safe to perform the *in vivo* efficacy tests. In any case, none of the peptide concentrations used, could significantly decrease the bacterial load in murine lungs. As weak effect was also observed with tobramycin, it is clear that factors other than proteolysis seem to inhibit the *in vivo* activity of the tested peptide. We indeed showed that the presence of murine BAL fluid markedly decreases, but does not abolish, the *in vitro* antimicrobial activity of *D*-BMAP18 against *P. aeruginosa* RP73. Murine BAL contains high amounts of lipids, mainly phospholipids, and a low amount of proteins (Goerke, 1998). It is known that lipids dispersed in aqueous solution induce α-helical AMPs to assume their amphipathic α-helix structures (Tossi et al., 2000). It is plausible that *D*-BMAP18 could be attracted mainly by surfactant phospholipids instead of bacterial lipopolysaccharide, forced to its active conformation by the lipids, and hence sequestered. Moreover, anionic mucin glycoproteins are also contained in BAL (Ballard and Inglis, 2004), which could potentially contribute to *D*-BMAP18 sequestration. In any case, the effects of BAL fluid on AMPs activity are still poorly understood and, in some cases, controversial. Some authors reported that the presence of BAL fluid did not interfere with antimicrobial activity of CaLL, an α-helical chimeric derivative of LL-37 and Cecropin A (Morris et al., 2012) with similar size to BMAP18. Apparently, this finding is in contrast with our observations, even though the different concentrations used (respectively 100 μg/ml vs. 8–16 μg/ml) do not allow a direct comparison between data. Conversely, Forde et al. showed that BAL fluid negatively interferes with bactericidal activity of different host defense peptides pro-drug (Forde et al., 2014), but the antimicrobial activity could be partially restored by increasing the ionic force of the medium. Interestingly, we observed a similar behavior using *D*-BMAP18. We previously observed that BMAP peptides, in contrast to most AMPs, continue to be effective also in hypertonic buffers (unpublished), and here demonstrated that the addition of 300 mM NaCl to BAL is favorable for the activity of *D*-BMAP18. High salts concentrations possibly counteracted the putative electrostatic sequestration of the peptide by BAL fluid components, as also previously suggested (Forde et al., 2014, 2016) and could open the possibility to use this molecule also in combination with hypertonic saline solutions, already used in the clinic (Reeves et al., 2012).

In conclusion, we showed that *D*-BMAP18 is an effective AMP against CF-related Gram-negative pathogens, being stable in biological fluid such as murine BAL, and not cytotoxic at low micromolar concentrations. However, we also demonstrated that, despite these desirable properties, *D*-BMAP18 is not yet suitable for *in vivo* applications, requiring additional studies for its optimization and lung delivery.

In this study we highlighted some critical points that should be addressed for designing AMPs suitable for pulmonary infections: (i) peptides are often prone to proteolytic digestion in the lungs, and this problem should not be underestimated regardless of the structural diversity of the studied peptide from that of other peptides known to be degraded; (ii) the composition of pulmonary fluid plays an important role: under these conditions, the AMP should maintain its specificity for bacteria and its antimicrobial potential; (iii) the assessment of the protective activity in a pulmonary infection model is a crucial step to subsequently focus on other variables that could determine the success of the peptide. These considerations may help drawing a route to have more chances in obtaining effective compounds for the fight against antibiotic-resistant pathogens.

## Author contributions

MM and MD synthesized the peptide, performed the microbiological and biochemical experiments *in vitro*, MD, GR and SP performed the cytotoxicity experiments. AP and GDB performed the *in vivo* toxicity assay and protective studies. MM, MS, AP, GDB and MD wrote and edited the manuscript. MM and MS designed the experiments. MS supervised the whole project.

### Conflict of interest statement

The authors declare that the research was conducted in the absence of any commercial or financial relationships that could be construed as a potential conflict of interest.
