# Supplementary material for: D-BMAP18 Antimicrobial Peptide Is Active In vitro, Resists to Pulmonary Proteases but Loses Its Activity in a Murine Model of Pseudomonas aeruginosa Lung Infection
Source: Front Chem. 2017 Jun 19;5:40. doi: 10.3389/fchem.2017.00040 (PMC5474674; doi:10.3389/fchem.2017.00040)
Supplement: Supplementary file 1 [file DataSheet1.PDF]

D-BMAP18 antimicrobial peptide is active *in vitro*, resists to pulmonary proteases but loses its activity in a murine model of *Pseudomonas aeruginosa* lung infection.

Mario Mardirossian[1,4#], Arianna Pompilio[2,3#], Margherita Degasperi[1], Giulia Runti[1], Sabrina Pacor[1], Giovanni Di Bonaventura[2,3], Marco Scocchi[1*].

[1]Department of Life Sciences, University of Trieste, Trieste, Italy

[2]Department of Medical, Oral, and Biotechnological Sciences, "G. d'Annunzio" University of Chieti-Pescara, Chieti, Italy

[3]Center of Excellence on Aging and Translational Medicine (CeSI-MeT), "G. d'Annunzio" University Foundation, Chieti, Italy

# M. Mardirossian and A. Pompilio equally contributed to the work.

Correspondence: corresponding author Marco Scocchi (mscocchi@units.it)

In vivo acute toxicity of D-BMAP18

Compared to control unexposed mice, exposure to peptide caused a significant (p<0.01) decrease in weight during the first 2 days regardless of doses used (Figure S1). This effect persisted until day 3 p.e. when peptide was administered at 2, 4, and 8 mg/kg (p<0.05), and until day 4 p.e. at 4 and 8 mg/kg (p<0.05). The reduction caused by exposure to peptide at 4 mg/kg persisted until day 5 p.e. (p<0.01). Mice started to gain their weight after day 2 p.e. regardless of doses administered, although they were not able to completely get the initial body weight until day 5 p.e.. Variations in pulmonary weight confirmed the same trend found for macroscopic score analysis (Figure S2).

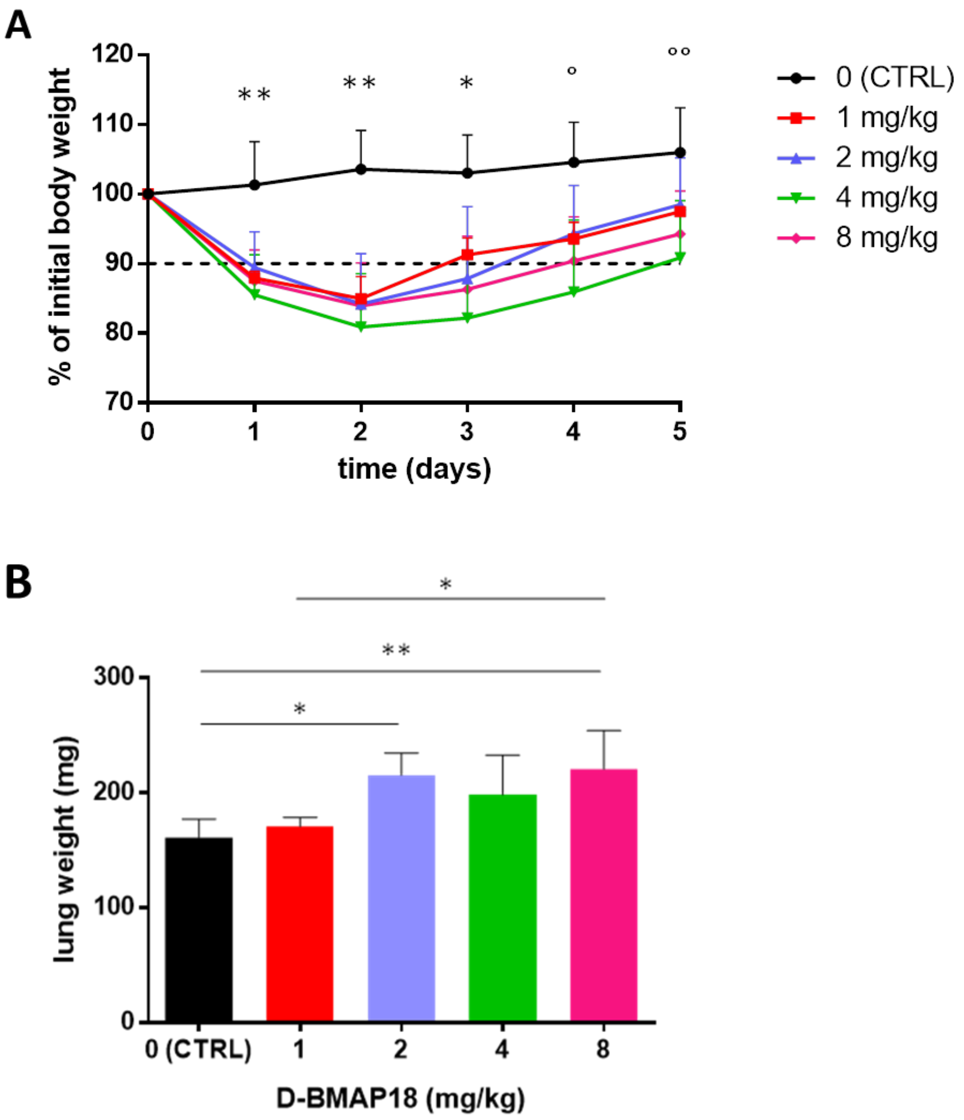

Figure S1. A) Changes in weight were graphed as the percentage of the body weight before single exposure to peptide. Results are shown as mean + SD values. Statistical analysis was carried out by ANOVA + Tukey's multiple comparison post-test: ***p*<0.01, CTRL vs other groups; **p*<0.05, CTRL vs (2, 4, and 8 mg/kg); °*p*<0.05, CTRL vs (4, and 8 mg/kg); °°*p*<0.01, CTRL vs 4 mg/kg. **B)** Lungs were removed en bloc from the chest via sterile excision, and immediately weighted. Results are shown as mean + SD values. *p<0.05, **p<0.01, ANOVA + Tukey's multiple comparison post-test.

***In vivo* antimicrobial activity of D-BMAP18**

The lungs of mice treated with D-BMAP18 1 and 2 mg/kg were slightly more edematous, as suggested by the observed weight, compared to those administrated with tobramycin 10 mg/kg (Figure S2).

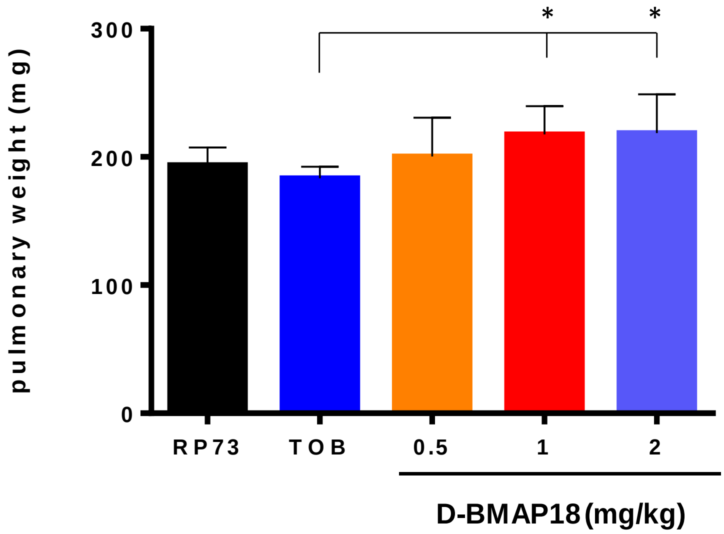

Figure S2. C57BL/6NCrl lungs were weighted on day 1 following *P. aeruginosa* RP73 infection. Results are mean + SD. *$p<0.05$, ANOVA + Newman-Keuls multiple comparison post-test.
